# Association between coffee consumption habits and non-alcoholic fatty liver disease in community-dwelling populations: data from the National Health and Nutrition Examination Survey 2013–2018

**DOI:** 10.1017/S1368980026101918

**Published:** 2026-02-02

**Authors:** Penglin Chen, Shiyan Wang, Tao Zhang, En Qiang Chen

**Affiliations:** 1 Department of Pharmacy, West China Hospital, Sichuan University, Chengdu 610041, China; 2 Center of Infectious Diseases, West China Hospital, Sichuan University, Chengdu 610041, China

**Keywords:** Coffee, Non-alcoholic fatty liver disease (NAFLD), Metabolic dysfunction-associated steatotic liver disease (MASLD), NHANES, Cross-sectional study

## Abstract

**Objective:**

Existing evidence suggests a potential association between coffee consumption and non-alcoholic fatty liver disease (NAFLD, now known as MASLD), yet the nature of this relationship remains ambiguous. The primary objective of this study was to comprehensively investigate and clarify the association between coffee intake and the occurrence of NAFLD.

**Design:**

A cross-sectional study design was employed, analysing data from National Health and Nutrition Examination Survey (NHANES) spanning from 2013 to 2018. Weighted univariate and multivariate logistic regression models were utilised to assess the relationship between coffee consumption and NAFLD. Restricted cubic spline analysis was conducted to explore any potential nonlinear associations. Forest plots were generated to visualise the impact of coffee consumption on NAFLD across different subgroups, and threshold effect analysis was performed to evaluate the nonlinear relationship between coffee consumption and NAFLD prevalence specifically in women.

**Setting:**

Data were from the US – representative NHANES.

**Participants:**

8062 subjects aged ≥ 20 years were included.

**Results:**

The weighted prevalence of NAFLD among the participants was 44·18 %. After controlling for confounding variables, coffee consumption was found to be negatively associated with the risk of NAFLD (OR = 0·96, 95 % CI: 0·94, 0·99). The association between coffee consumption and NAFLD was observed to vary by gender and education level. For the prevention of NAFLD in women, the optimal coffee intake was determined to be two cups.

**Conclusions:**

Increasing coffee intake emerges as a potentially effective non-pharmacological strategy for the prevention and management of NAFLD. Notably, for women, consuming two cups of coffee appears to represent the optimal threshold for maximising this beneficial effect.

Non-alcoholic fatty liver disease (NAFLD, now known as MASLD) is defined by the presence of macrovesicular steatosis in 5 % of hepatocytes, in the absence of other identified cause of steatosis (e.g. medications, starvation and monogenic disorders) in individuals who drink little or no alcohol (defined as < 20 g/d for women and < 30 g/d for men)^(1)^, which is the most prevalent chronic liver disease, with a global prevalence of about 25–32 %^(2)^. NAFLD has the potential to progress to severe liver conditions, including liver cirrhosis, end-stage liver disease, and hepatocellular carcinoma. It can also contribute to non-hepatic-related complications, such as cardiovascular and chronic kidney diseases^(3)^. Due to its asymptomatic nature in the early stages, many patients are diagnosed only when the disease has progressed to advanced stage^(4)^. leading to an escalating burden from NAFLD and its associated sequelae^(5)^. Therefore, the identification of biomarkers and risk factors for NAFLD, along with the development of preventive strategies, is critical for early detection and the reduction of disease progression. Obesity, insulin resistance, and metabolic syndrome are well-established risk factors for NAFLD^(6)^. Moreover, modifiable risk factors such as physical activity and diet may serve as the basis for intervention strategies aimed at preventing or treating NAFLD^(7)^. Both dietary nutrition and exercise play significant roles in the improvement of non-alcoholic fatty liver disease (NAFLD). Relevant studies have shown that a reasonable diet structure and nutritional intervention can effectively regulate liver lipid metabolism, reduce liver inflammatory responses and the degree of fibrosis, and thereby promote the reversal and disease control of NAFLD. Meanwhile, the combination of exercise and dietary intervention can also promote liver fat phagocytosis through different pathways, further contributing to the improvement of NAFLD^(8,9)^. A prospective cohort study also showed that a proinflammatory diet was associated with an increased risk of severe NAFLD, independent of confounding factors such as metabolic syndrome. Therefore, it is necessary to learn more about dietary factors that may help prevent and manage NAFLD.

Coffee, a widely consumed beverage, is favoured for its unique aroma and taste^(10)^. It is a complex mixture of biologically active compounds, including kahweol, cafestol and caffeine. Caffeine^(11)^, a natural stimulant found in coffee, tea, soft drinks, and energy drinks, primarily exerts its biological effects by competitively antagonising adenosine receptors^(12)^. Many studies have attributed these beneficial health effects of coffee to its caffeine content. Numerous studies have demonstrated that coffee consumption can offer health benefits on the liver, including a reduced risk of fatty liver disease and a lower severity of hepatic steatosis. Additionally, coffee consumption has been shown to significantly reduce the risk of type 2 diabetes mellitus and metabolic syndrome^(13)^. In a large population-based study, coffee drinkers at high risk for liver injury exhibited lower levels of aminotransferase activity compared with non-coffee drinkers^(14)^. Regular coffee consumption has also been linked to alleviation of hepatic fibrosis in patients with NAFLD^(15)^. However, the reduction in hepatic fibrosis appears to be specific to coffee and is not observed with other caffeinated beverage, such as tea and cocoa^(16)^. Therefore, coffee is regarded as a potential non-pharmacological intervention for the primary and secondary prevention of NAFLD^(17)^. However, the amount and form of coffee that must be consumed to achieve this hepatoprotective effect has not been determined^(18)^.

Currently, large-sample epidemiological studies investigating the relationship between coffee consumption and NAFLD are limited. Therefore, further research is needed to explore the potential beneficial effects of coffee consumption in relation to NAFLD. This study, for the first time, assessed the association between coffee consumption and NAFLD risk using data from NHANES (2013–2018). Our findings will provide valuable insights for future basic and clinical research, as well as offer new suggestions for nutritional guidelines and health policy.

## Materials and methods

### Study population

**Figure 1.**
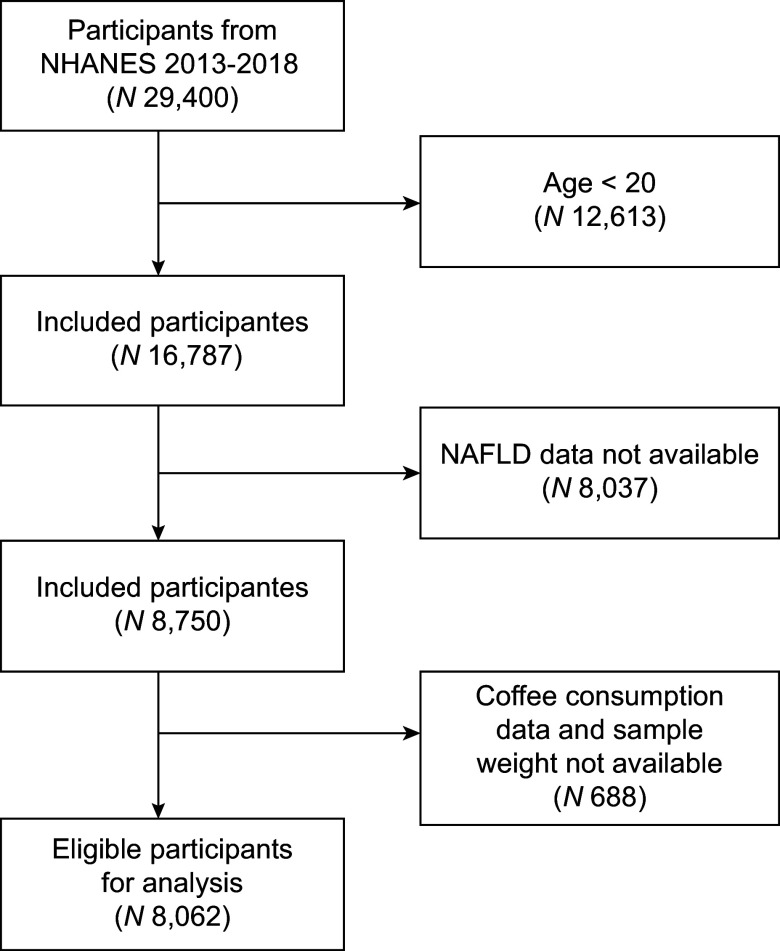
Diagram showing how the study population is selected.

Data for this study were obtained from the NHANES database, an annual survey conducted by the Centers for Disease Control and Prevention in the USA, which uses a national, multistage, stratified cluster design to represent the noninstitutionalised civilian population. Participants provided written informed consent to the Ethics Review Board of the National Center for Health Statistics. We selected data from NHANES (2013–2018), which recruited 29 400 participants. In our study, we excluded participants who are younger than 20 years (*n* 12 613), those without NAFLD data (*n* 8037) and individuals with missing coffee consumption data or sample weight (Figure 1) Hepatitis B Virus (HBV)/Hepatitis C Virus (HCV) infection is a key cause of liver disease, independently inducing hepatocellular injury with a pathological mechanism distinct from MASLD. Finally, 8062 eligible participants were included in the study.

### Exposure variable

Coffee intake was measured using FFQ collected by NHANES from 2013 to 2018, along with data on food, beverages and dietary supplements consumed during two 24-h dietary periods (midnight to midnight). As described in previous studies, coffee consumption in NHANES was associated with a specific eight-digit food code (code beginning with 921) in the Food and Nutrient Database for Dietary Studies^(17)^. Participants with two 24-h dietary recalls were selected, and average coffee consumption from these two recalls was used. In this study, one cup of coffee was defined as 283·5 g^(19)^. In addition, coffee intake was further categorised into four groups: 0, < 2, 2–4 and > 4 cups per day.

### Outcome variable

NAFLD status was assessed using the self-report questionnaire and NAFLD liver score, developed by the Department of Medicine and Minerva Medical Research Institute at Helsinki University^(20)^. This score formula was derived using a multivariate logistic regression model incorporating factors such as metabolic syndrome, type 2 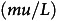 diabetes, fasting insulin (fS), serum aspartate aminotransferase ratio and aspartate aminotransferase to serum alanine aminotransferase ratio. The formula for the NAFLD liver fat score is as follows: *NAFLD liver fat score* = −0·89 + 1·18 × *metabolic syndrome* (*yes* = 1/*no* = 0) + 0·45 × *diabetes* (*yes* = 2/no = 0) + 0·15 × *fS* - *insulin* + 0·04 × *fS* - *AST* (*U/L*) −0·94 × *AST/ALT*. Participants with a score > −0·640 were classified as having NAFLD, based on the optimal cut-off point^(21)^. Documents detailing data collection and laboratory testing methods are publicly available on the NHANES website. It should be noted that this study did not use imaging methods (e.g. ultrasound, MRI) or liver biopsy (the gold standard for NAFLD diagnosis) due to the population-based cross-sectional design of NHANES, which prioritises non-invasive, large-scale feasible assessment tools. The combination of self-report and the NAFLD Liver Fat Score was chosen to strike a balance between diagnostic accuracy and practicality in a large sample (*n* 8062) setting, consistent with methodological practices of previous NHANES-based NAFLD studies.

### Covariates

Covariates for this study included age, sex, race, marital status, family income to poverty ratio (FIR), education, BMI, smoke status, DM, hypertension and CVD. Race of participants was divided into five categories: Mexican-American, non-Hispanic white, non-Hispanic black and other Hispanic. A low-income level was defined as FIR < 1·3^(22)^. BMI group is designated as underweight or normal weight (< 25·0 kg/m^2^), overweight (25·0–29·9 kg/m^2^) and people living with obesity or individuals with obesity ( 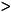 29·9 kg/m^2^)^(23)^. DM was defined as an individual must have glycated Hb of at least 6·5 %, fasting blood glucose of 126 mg/dl, 2-h plasma glucose of at least 200 mg/dl and be taking an insulin or hypoglycaemic medication currently^(24)^. Hypertension was defined as participants with the systolic blood pressure ≥ 140 mmHg or the diastolic blood pressure ≥ 90 mmHg and/or self-reported doctor diagnosis and/or antihypertensive treatment^(25)^. Among the CVD identified were congestive heart failure, CHD, angina, heart attack and stroke.

### Statistical analysis

According to the NHANES analysis guidelines, sample weights were used for all data. Descriptive statistics, including mean, median, sd, range and quartiles, were used for continuous variables, while frequency tables were used for categorical variables. Non-normally distributed continuous variables were summarised using median and interquartile range. Differences in the characteristics between groups were assessed using the *t* test for continuous variables, the Wilcoxon rank sum test for complex sampling and the Rao–Scott χ2 test for the weighted percentages of categorical variables, providing a comprehensive overview of the study population. To examine the association between coffee consumption and NAFLD, we performed weighted univariate and multivariate logistic regression analysis, following the Strengthening the Reporting of Observational Studies in Epidemiology^(26)^. Three models were developed: model 1 (unadjusted); model 2 (adjusted for age, sex and race) and model 3 (adjusted for age, sex, race, Marital status, FIR, education, CVD, excessive drinking and smoke status). To further investigate the dose–effect relationship between coffee consumption and NAFLD, restricted cubic spline analysis was employed. Forest plots were used to visualise the robustness of coffee consumption to NAFLD in different subgroups. Threshold effect analysis was used to assess the nonlinear relationship between coffee consumption and NAFLD prevalence in the female population.

We conducted a causal mediation analysis mediated by WHtR: exposure was the number of coffee cups per day, the outcome was NAFLD (NAFLD-LFS > −0·64) and the covariates were the same as those in the main model. Totally, 100 simulations were conducted using R’s mediation (v4.5.0) within the Quasi-Bayesian framework to estimate Average Causal Mediation Effect (ACME), Average Direct Effect (ADE), Total Effect (TE) and their 95 % CI.

## Results

### Basic characteristics of the population

Table 1 exhibits the basic characteristics of the 8062 participants included in the study. Report baseline distribution by survey weight and SMD_Unadj/SMD_IPTW (see online supplementary material, Supplemental STable 1).The median age was 46 years, with 48·30 % of participants being males and 51·70 % females. Our analysis found that participants with NAFLD were more likely to be male, Mexican American and married. In addition, those participants were more likely to be people living with obesity or individuals with obesity, smoking and have a higher prevalence of DM, hypertension and CVD. Of note, coffee consumption was significantly different between participants with and without NAFLD (*P* = 0·002). However, there were no significant differences between the two groups in terms of FIR and education level.

**Table 1.** Weighted patient demographics and baseline characteristics

Characteristic	NAFLD						
			0		1		
	Overall, *n* 324 116 079^*^		Weighted *n* 183 446 291, Unweighted *n* 4500^*^		Weighted *n* 140 669 788, Unweighted *n* 3562^*^		
	*n*	%	*n*	%	*n*	%	*P* value^†^
Age							
Median	46		39		53		< 0·001
IQR	28, 60		25, 56		36, 64		
Sex							0·001
Male	3955	48·30 %	2104	44·40 %	1851	53·40 %	
Female	4107	51·70 %	2396	55·60 %	1711	46·60 %	
Race							0·025
Mexican American	1297	9·77 %	617	8·43 %	680	11·51 %	
Non-Hispanic White	2998	62·91 %	1707	64·02 %	1291	61·46 %	
Non-Hispanic Black	1713	11·38 %	970	11·52 %	743	11·19 %	
Other Hispanic	844	6·28 %	441	6·17 %	403	6·43 %	
Other race – Including multi-racial	1210	9·65 %	765	9·85 %	445	9·41 %	
Coffee consumption (cups/d)							
Median	0·00		0·00		0·00		0·002
IQR	0·00, 2·01		0·00, 1·78		0·00, 2·03		
Marital status							< 0·001
Never married	2618	26·44 %	1802	32·86 %	816	18·07 %	
Married/Living with Partner	4001	56·88 %	2019	52·81 %	1982	62·19 %	
Widowed/Divorced/Separated	1443	16·68 %	679	14·33 %	764	19·74 %	
FIR							0·428
˂ 1·3	2709	24·17 %	1497	23·49 %	1212	25·06 %	
1·3–3·5	3137	36·64 %	1720	36·35 %	1417	37·01 %	
˃ 3·5	2216	39·19 %	1283	40·15 %	933	37·93 %	
Education							0·319
˂ High school	2626	20·78 %	1543	21·43 %	1083	19·93 %	
High school	1665	22·52 %	887	21·56 %	778	23·77 %	
˃ High school	3771	56·70 %	2070	57·01 %	1701	56·30 %	
BMI							< 0·001
≤ 24·9	2750	32·55 %	2397	50·37 %	353	9·32 %	
25–29·9	2402	30·33 %	1337	31·98 %	1065	28·18 %	
≥ 30	2910	37·12 %	766	17·66 %	2144	62·50 %	
Smoke status							< 0·001
No	4875	58·07 %	2878	61·87 %	1997	53·11 %	
Yes	3187	41·93 %	1622	38·13 %	1565	46·89 %	
DM							< 0·001
No	6539	84·82 %	4315	97·26 %	2224	68·60 %	
Yes	1523	15·18 %	185	2·74 %	1338	31·40 %	
Hypertension							< 0·001
No	5050	63·76 %	3458	80·20 %	1592	42·32 %	
Yes	3012	36·24 %	1042	19·80 %	1970	57·68 %	
CVD							< 0·001
No	7271	91·05 %	4261	95·56 %	3010	85·15 %	
Yes	791	8·95 %	239	4·44 %	552	14·85 %	

NAFLD, non-alcoholic fatty liver disease.

The analysis included measures such as mean, median, sd, range and quartiles for continuous variables and frequency tables for categorical variables. Nonnormally distributed continuous variables were described using median and interquartile range (IQR). The analysis was conducted using complex sampling weights to ensure representativeness of the study population.

To examine variations in variable characteristics among groups, we employed *t* test and survey Wilcoxon rank-sum test for complex survey samples to continuous variables and the Rao–Scott *χ* ^2^ test for weighted percentages of categorical variables, comprehensively describing the entire population.

* Median (IQR); *n* (unweighted) (%).

† Wilcoxon rank-sum test for complex survey samples; *χ* ^2^ test with Rao and Scott’s second-order correction.

### Logistic regression analysis of coffee consumption and non-alcoholic fatty liver disease

**Figure 2.**
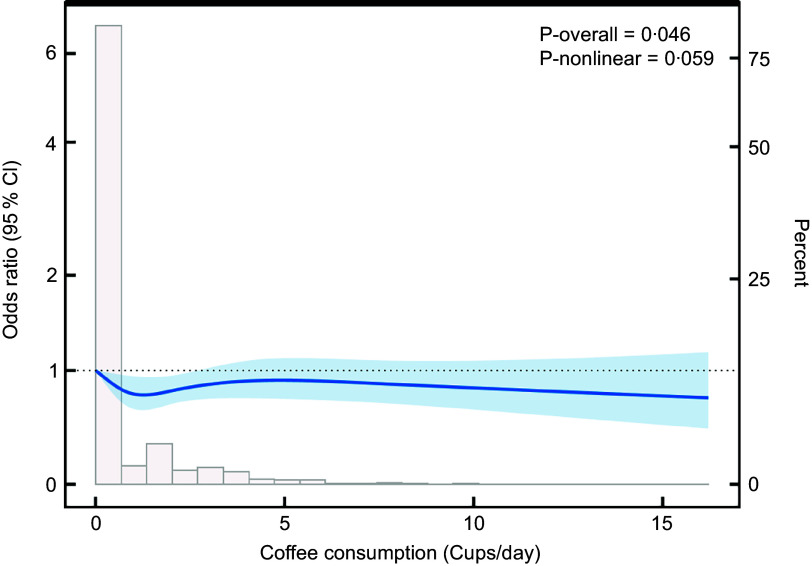
Dose–response relationship of coffee intake (cups per day) with the risk of occurrence of MAFLD. NAFLD, non-alcoholic fatty liver disease.

We used multiple logistic regression models to validate the relationship between coffee intake and NAFLD, and the results are shown in Table 2. The marginal OR of Inverse Probability of Treatment Weighting (IPTW) + dual robustness was used as the supplementary result of the sensitivity analysis (see online supplementary material, Supplemental STable 2). In the unadjusted crude model (model 1), coffee intake was significantly positively associated with the risk of NAFLD when treated as a continuous variable (OR = 1·03, 95 % CI: 1·01, 1·05, *P* = 0·003). This suggests a 3 % increase in the risk of NAFLD for each additional cup of coffee consumed. After full adjustment for confounding factors, the positive association between coffee intake and NAFLD risk changed to a negative association (OR = 0·96, 95 % CI: 0·94, 0·99, *P* = 0·002), which means that each cup of coffee consumption was associated with a 4 % reduction in the risk of NAFLD (model 3). To verify the stability of the results, coffee intake was further divided into four groups: 0, < 2, 2–4 and > 4 cups/d and validated in three models. In model 1, NAFLD risk was positively associated with coffee intake in all groups. However, after adjusting for confounding factors, coffee intake was negatively associated with NAFLD risk in each group (OR = 0·86, 95 % CI: 0·75, 0·98; OR = 0·87, 95 % CI: 0·76, 1·00; OR = 0·85, 95 % CI: 0·75, 0·98). Compared with non-coffee drinkers, the risk of NAFLD was significantly reduced by 21 % (OR = 0·79, 95 % CI: 0·64, 0·96, *P* = 0·002) in the group consuming > 4 cups/d after fully adjusting for confounding factors. To further explore the relationship between coffee intake and NAFLD, we performed a dose–response diagram of restricted cubic spline was added, presenting the overall *P* and nonlinear *P* (Figure 2). No nonlinear association was found between coffee consumption and NAFLD (*P* value = 0·008, *P* nonlinear = 0·685), which further supports the presence of an inverse linear relationship.

**Table 2.** Association between coffee consumption and NAFLD

Characteristic	Model 1			Model 2			Model 3		
	OR^*^	95 % CI^*^	*P* value	OR^*^	95 % CI^*^	*P* value	OR^*^	95 % CI^*^	*P* value
Coffee consumption (cups/d)									
0	–	–		–	–		–	–	
˂ 2	1·25	1·10, 1·41	< 0·001	0·86	0·75, 0·98	0·027	0·97	0·83, 1·14	0·734
2–4	1·32	1·17, 1·49	< 0·001	0·87	0·76, 1·00	0·047	0·89	0·76, 1·04	0·143
˃ 4	1·37	1·17, 1·59	< 0·001	0·85	0·75, 0·98	0·034	0·79	0·64, 0·96	0·019
Continuous	1·03	1·01, 1·05	0·003	0·98	0·96, 1·00	0·045	0·96	0·94, 0·99	0·002

* OR and CI.

Model 1: no covariates were adjusted.

Model 2: adjusted for age, sex and race.

Model 3: adjusted for age, sex, race, marital status, FIR, education, BMI, DM, hypertension, CVD and smoke status.

### Subgroup analysis of the association between coffee consumption and non-alcoholic fatty liver disease

**Figure 3.**
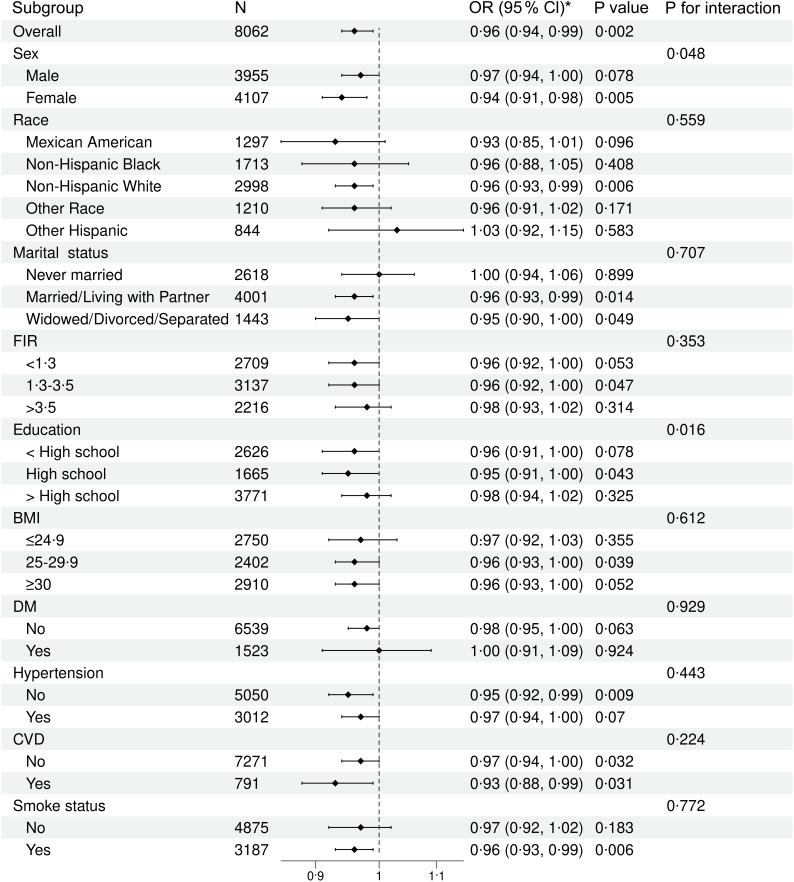
Subgroup analysis of the association between coffee consumption and NAFLD. NAFLD, non-alcoholic fatty liver disease.

As seen in Figure 3, subgroup analysis was performed to evaluate the robustness of association between coffee consumption and NAFLD. The interactions with sex, race, marital status, FIR, education, BMI, DM, hypertension, CVD and smoke status were also tested to evaluate that if there was any significant dependence of the effect modifier on this relationship. The results showed an interaction between sex and education subgroups (*P*_for interaction_ < 0·05). Interestingly, the negative association between coffee consumption and NAFLD prevalence risk was significantly stronger among participants who were female and had less education. In contrast, no significant interaction was observed in the other subgroups, indicating that the relationship was stable.

**Figure 4.**
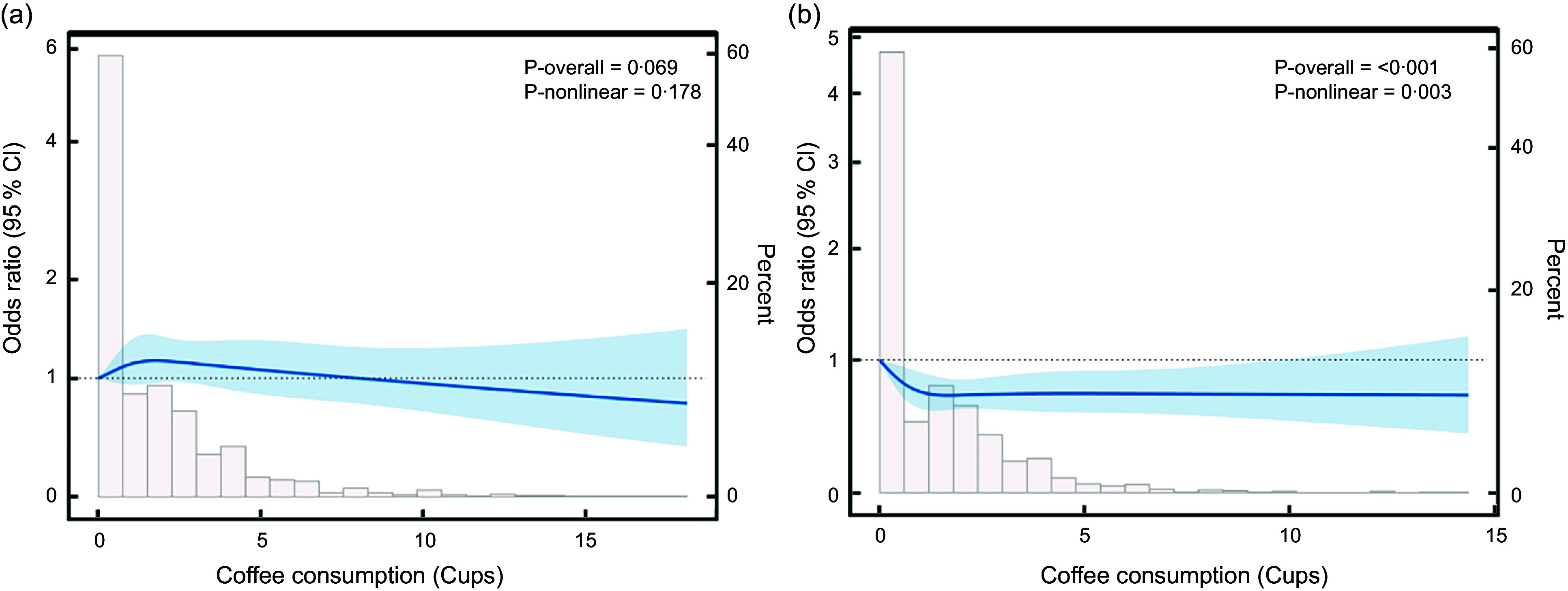
Dose–response relationship between coffee consumption and NAFLD prevalence by sex: (a) Male subgroup and (b) female subgroup. NAFLD, non-alcoholic fatty liver disease.

Figure 4 further presents the sex-specific association between coffee consumption and NAFLD risk. In this study, we found a linear relationship between coffee consumption and NAFLD prevalence in men. However, in women, coffee consumption showed a U-shaped non-linear relationship with NAFLD prevalence. By further threshold effect analysis (as shown in Table 3), this study found that the prevalence of NAFLD was relatively lowest when coffee consumption reached two cups in women. Higher coffee intake did not further reduce NAFLD prevalence.

**Table 3.** Threshold effect analysis of coffee consumption on NAFLD

	OR^*^	95 % CI	*P* value
Fitting by standard logistic regression model	0·96	0·94, 0·99	0·002
Fitting by piecewise logistic regression model (break-point = 2)			
Coffee consumption (cups) < 2	0·84	0·77, 0·92	< 0·001
Coffee consumption (cups) ≥ 2	1·00	0·96, 1·05	0·841
Log likelihood ratio			**0·002**

NAFLD, non-alcoholic fatty liver disease.

* Adjusted for age, sex, race, marital status, FIR, education, BMI, hypertension, CVD and smoke status.

### Sensitivity analysis of the association between coffee consumption and non-alcoholic fatty liver disease

After adding physical activity and aMED to the main model, the negative dose–response trend of coffee intake and NAFLD remained. The morphology and significance of the classification dose (< 2, 2–4, > 4 *v*. 0 cup/d) and restricted cubic spline curve did not undergo substantial changes. Physical activity was associated with both aMED and NAFLD in the expected direction, but had a limited effect on the estimation of coffee-NAFLD (see the sensitivity analysis table).

The total effect of coffee consumption on NAFLD was statistically significant (coefficient = 0·00636, 95 % CI: 0·00223, 0·00990, *P* = 0·020). The indirect effect through WHtR was significant (coefficient = 0·00922, 95 % CI: 0·00699, 0·01179, *P* < 0·001). The direct effect of coffee consumption on NAFLD was NS (coefficient = −0·00286, 95 % CI: −0·00672, 0·00051, *P* = 0·120). The total effect proportion mediated by WHtR was 142·7 % (95 % CI: 89·1 %, 325·5 %).

## Discussion

The results show that WHtR is a significant mediating variable in the relationship between coffee consumption and NAFLD. Significant indirect effects suggest that coffee consumption may mainly influence the risk of NAFLD through its association with WHtR. The insignificance of the direct effect implies that the relationship is largely explained by the mediating variables. A mediating ratio exceeding 100 % may indicate the presence of inhibitory effects or other mediating pathways. These findings emphasise the importance of considering body composition indicators (such as WHtR) when studying dietary factors and liver health. Given the limitations of cross-sectional design and proxy outcomes/mediating measurements, the mediating role of WHtR should be interpreted with caution; However, its significance supports that ‘body fat distribution (visceral fat) may be an important pathway for the association between coffee and hepatic fatty diseases’.

The U-shaped coffee–NAFLD relationship in women is supported by sex-stratified studies and aligned with meta-analytic evidence (which also explains null associations via methodological flaws or high-risk populations). The interaction between coffee and education further reflects socio-economic modulation of this biological pattern – with less educated women deriving greater benefit from optimal coffee intake due to higher baseline dietary and metabolic risk. Future studies should stratify by menopausal status, collect detailed coffee preparation data and adjust for dietary inflammatory markers to fully validate these mechanisms – and address the current study’s limitations of unstratified female subgroups and lack of coffee type data.

An investigation of the relationship between coffee consumption and NAFLD was undertaken in this study. Results showed coffee consumption is negatively related to NAFLD (OR = 0·96, 95 % CI: 0·94, 0·99, *P* = 0·002). By increasing coffee consumption each 1 cups/d, the risk of NAFLD was reduced by 4 %, suggesting that coffee consumption may be a potential protective factor for NAFLD, but a causal relationship could not be established. In addition, the result of unadjusted model indicated that coffee consumption was positively associated with the risk of NAFLD, which is worth further exploring the reasons.

CVD is a major cause of mortality in patients with NAFLD^(27)^. Disruption in lipoprotein metabolism, endothelial function, the increased presence and higher-risk nature of atherosclerotic lesions and impaired ischaemic compensatory mechanisms support the relationship between NAFLD and CVD^(28)^. Hypertension is also closely associated with NAFLD, with a bidirectional relationship between the two conditions^(29)^. A study conducted in non-hypertensive individuals indicated that elevated blood pressure could also serve as a risk factor for NAFLD^(30)^. Caffeine, a known stimulant, has been shown to enhance the forearm blood flow response to acetylcholine, an endothelium-dependent vasodilator, thus increasing both systolic and diastolic blood pressure. In vitro, it antagonises adenosine receptors to reduce hepatocyte oxidative stress, inhibit pro-inflammatory factor (e.g. TNF-*α* and IL-6) release and suppress TGF-*β* induced Connective Tissue Growth Factor (CTGF) expression via PPARγ/SMAD2/3 pathways (anti-fibrosis), indirectly alleviating lipid deposition^(31)^. Although coffee may raise blood pressure in the short term, it does not increase risk for long-term hypertension. Conversely, moderate coffee consumption has potentially beneficial effects on cardiometabolic health^(32)^. This effect may partly explain the consistency of our findings.

Obesity and smoking are well-established risk factors for NAFLD. Tobacco contains a variety of harmful substances, particularly nicotine, which, when combined with a high-fat diet, can exacerbate hepatocyte apoptosis and promote the development of NAFLD^(33)^. Smokers living with obesity or individuals with obesity were more likely to suffer from NAFLD due to the synergistic effects of these factors. Interestingly, a study found that coffee consumption could mitigate nicotine-induced NAFLD in combination with a high-fat diet by reducing lipid accumulation, regulating hepatic lipid metabolism, alleviating oxidative stress, attenuating inflammation, and restoring liver function^(34)^. It is essential to emphasise that the detrimental impact of smoking on health is multifaceted. Smoking cessation and slimming remain the effective strategy for averting the related NAFLD complications and reducing the risk of mortality.

In our subgroup analysis, we found that there was a significant interaction between gender and education for the negative association between coffee intake and NAFLD, with the negative association between coffee intake and NAFLD risk being significantly stronger among women and participants with lower education. For the prevention of NAFLD, the optimal daily dose of coffee intake for women is two cups. Studies have shown that there are differences in the prevalence and severity of NAFLD between men and women at different ages, and for women, oestrogen may play a protective role^(35)^. Moreover, observations in animal models suggest that males may have more severe steatosis and steatohepatitis, more proinflammatory/profibrotic cytokines and a higher incidence of liver tumours than females^(36)^. The effect of education on NAFLD is thought to be positive^(37)^. Currently, there is no evidence of an association between education level and NAFLD. This may be due to the fact that these less educated populations are more likely to choose coffee, and the protective effect of coffee on reducing the risk of NAFLD is more pronounced in this population. In addition, the amplified coffee effect in less educated individuals is supported by existing literature on socioeconomic disparities in diet and NAFLD risk and can be further validated through targeted hypotheses. Future studies incorporating detailed dietary data, coffee preparation methods and baseline risk assessments will help confirm these mechanisms^(38)^.

NAFLD is closely associated with reduced insulin sensitivity, which plays a central pathogenic role in the development of type 2 diabetes mellitus. Patients with type 2 diabetes mellitus have a higher prevalence of NAFLD, ranging from 30 % to 75 %^(39)^. Hyperglycaemia significantly enhances the lipolysis in adipose tissue, leading to an increased release of free fatty acids into the bloodstream. These free fatty acids then enter the liver, causing ectopic lipid deposition and accelerating the progression of NAFLD^(40)^.

Many studies have highlighted the hepatoprotective effect of coffee, which may be attributed to both caffeine and non-caffeine compounds^(41)^. Caffeine had been shown to reduce oxidative stress and liver inflammation in *in vitro* study with indications of anti-fibrotic properties in hepatocytes^(42)^. However, non-caffeine components also play a crucial role in liver protection^(43)^. For example, antioxidants such as chlorogenic acid and uridine diphosphate glucuronosyltransferase can prevent lipid accumulation in hepatocytes, reduce the inflammatory response and improve insulin sensitivity^(44)^. Additionally, diterpenes such as cafestol and kahweol exert antioxidant effects, protecting the liver by suppressing inflammatory reactions and reducing the expression of inflammatory markers. While the exact mechanism of this effect remains unclear, it is clear that the potential hepatoprotective benefit of coffee needs to be further investigated.

Therefore, for patients diagnosed with NAFLD, combining dietary changes with exercise, especially increasing coffee intake, may help reduce liver fat and consequently lower the risk of NAFLD-related complications. This approach is crucial in the daily management of NAFLD patients. The findings of this study highlight the complex relationship between coffee consumption and NAFLD. To gain a deeper understanding of this association, further research is needed across diverse populations and to explore the underlying biological mechanisms. Additionally, we emphasise the importance of the Mediterranean diet and regular exercise. It is worth noting that the effects of coffee consumption may vary across different populations.

This study has several limitations, despite utilising NHANES data, which provides broad, representative information. First, it is crucial to acknowledge a key methodological limitation of this study: the cross-sectional design, which inherently restricts our ability to establish a causal relationship between coffee consumption and NAFLD. Second, the use of a 24-h dietary recall method to collect coffee intake data, along with the self-reported diagnosis of NAFLD, may introduce recall bias. Inherent mismatch with habitual intake assessment: habitual coffee consumption reflects stable long-term behavior, but 24-h recalls only capture intake on specific days (e.g. weekdays *v*. weekends). This temporal narrowness fails to account for routine fluctuations (e.g. higher intake on workdays *v*. none on weekends), leading to over- or underestimation of usual intake. Additionally, short-term disruptions (e.g. illness and temporary schedule changes) during recall days can further distort measurements of long-term habits, weakening the study’s ability to establish a robust dose–response relationship between habitual coffee intake and NAFLD risk – particularly critical for subgroups like women, where the proposed optimal threshold (two cups/d) depends on precise habitual intake data. In future studies, we may consider using more objective measures, such as biomarker testing, to assess coffee intake, as well as combining clinical diagnostic criteria and imaging to determine the diagnosis of NAFLD, in order to reduce the impact of recall bias on study results. Third, the findings may not be generalisable to populations outside of the exclusion criteria, as we applied a series of specific exclusions in the analysis. In follow-up studies, we can try to include a wider population to improve the generality of the findings. Fourth, due to the complexity of confounding factors, we cannot rule out the influence of other potential confounders on the study’s results. These steps would further improve the robustness of NAFLD diagnosis in large-scale studies. Methods such as FFQ and biomarker testing better capture habitual intake, highlighting this study’s methodological gaps. Such discrepancies may bias the study’s conclusions: for example, underestimated high intake (e.g. 5 cups/d recorded as 4) could weaken the observed protective effect of > 4 cups/d against NAFLD. NHANES was unable to stably distinguish the brewing method of coffee (filtered and espresso/instant) and additives (sugar and cream) within the used period. In this study, the exposure was the total number of cups per day, and it was impossible to stratify by type/additive. The related misclassification was more likely to shift the effect towards zero. Future studies can further improve the control of confounders and include more potentially relevant factors for analysis so as to more accurately reveal the true relationship between coffee consumption and NAFLD. Finally, how coffee affects the pathogenesis of NAFLD has not been thoroughly investigated in this study. Future studies can combine FFQ (for long-term habit assessment) with 24-h recalls (to correct portion size bias). Incorporate biomarkers (e.g. urinary caffeine) to validate self-reported intake. Use prospective designs with repeated measurements to map individual coffee intake trajectories, enhancing the reliability of dose–response and subgroup threshold conclusions. Combine the NAFLD Liver Fat Score with objective biomarkers (e.g. serum cytokeratin-18, a non-invasive marker for hepatic steatosis), validate the score against MRI or liver biopsy in a subset of participants and use repeated measurements of metabolic covariates to reduce recall bias. Follow-up studies can strengthen the mechanism studies at the cellular and molecular levels and further clarify the protective mechanism of coffee against NAFLD.

### Conclusions

Coffee consumption is associated with a reduced risk of developing NAFLD, with potential variations based on population characteristics. Our findings suggest that increasing coffee intake could serve as a potential strategy for NAFLD management. Particularly in women, consuming approximately two cups of coffee per day may be optimal for reducing NAFLD risk, although further research is required to confirm this threshold.

## Supporting information

Chen et al. supplementary material 1 Chen et al. supplementary material

Chen et al. supplementary material 2 Chen et al. supplementary material

## Supplementary material

For supplementary material accompanying this paper visit https://doi.org/10.1017/S1368980026101918
