# Supplementary material for: Association between coffee consumption habits and non-alcoholic fatty liver disease in community-dwelling populations: data from the National Health and Nutrition Examination Survey 2013–2018
Source: Public Health Nutr. 2026 Feb 2;29(1):e35. doi: 10.1017/S1368980026101918 (PMC12951349; doi:10.1017/S1368980026101918)
Supplement: Chen et al. supplementary material 1 — Chen et al. supplementary material [file S1368980026101918sup001.docx]

STable1 The baseline distribution of the report by survey weight and SMD_Unadj/SMD_IPTW

| Variable | Level | 0 | <2 | ‘2-4’ | >4 | SMD_Unadj | SMD_IPTW |
| --- | --- | --- | --- | --- | --- | --- | --- |
| Age |  | 39 (19.6) | 48.1 (18.1) | 51.7 (16.7) | 54.6 (13.8) | 0.907740389 | 0.278432711 |
| BMI |  | 28.8 (7.8) | 28.9 (7.3) | 29 (6.8) | 28.9 (5.7) | 0.028696486 | 0.057484436 |
| Sex | Female | 52.70% | 62.80% | 48.40% | 40.80% | 0.445596282 | 0.020981382 |
|  | Male | 47.30% | 37.20% | 51.60% | 59.20% | 0.445596282 | 0.020981382 |
| Race | Mexican American | 11.60% | 11.50% | 8% | 3.90% | 0.720179459 | 0.168539447 |
|  | Non-Hispanic Black | 17.20% | 7.80% | 5.90% | 1.80% | 0.720179459 | 0.168539447 |
|  | Non-Hispanic White | 54.30% | 55.40% | 74% | 86.60% | 0.720179459 | 0.168539447 |
|  | Other Hispanic | 6% | 11.60% | 5.70% | 2.50% | 0.720179459 | 0.168539447 |
|  | Other Race - Including Multi-Racial | 11% | 13.80% | 6.30% | 5.20% | 0.720179459 | 0.168539447 |
| Smoke_status | No | 65.60% | 63.40% | 50.20% | 36.30% | 0.292493 | 0.061761 |
|  | Yes | 34.40% | 36.60% | 49.80% | 63.70% | 0.292493 | 0.061761 |
| Education | < High school | 27.40% | 17.30% | 13.20% | 11.20% | 0.434230423 | 0.139217751 |
|  | > High school | 49.40% | 61.10% | 65.70% | 65.90% | 0.434230423 | 0.139217751 |
|  | High school | 23.20% | 21.60% | 21.10% | 22.90% | 0.434230423 | 0.139217751 |
| Marital_status | Married/Living with Partner | 47.40% | 61.10% | 70.70% | 67.50% | 0.39513 | 0.124741 |
|  | Never married | 39.10% | 19.80% | 13.10% | 6.10% | 0.39513 | 0.124741 |
|  | Widowed/Divorced/Separated | 13.50% | 19% | 16.20% | 26.40% | 0.39513 | 0.124741 |
| Year | 2013-2014 | 32.80% | 27% | 29.70% | 30.40% | 0.183330132 | 0.050076476 |
|  | 2015-2016 | 35.20% | 32.20% | 33.50% | 34.10% | 0.183330132 | 0.050076476 |
|  | 2017-2018 | 32% | 40.80% | 36.70% | 35.50% | 0.183330132 | 0.050076476 |
| DM | No | 85.30% | 86% | 84.20% | 82.50% | 0.095794526 | 0.148923091 |
|  | Yes | 14.70% | 14% | 15.80% | 17.50% | 0.095794526 | 0.148923091 |
| Hypertension | No | 69.10% | 63.40% | 57% | 54.10% | 0.309352027 | 0.152101374 |
|  | Yes | 30.90% | 36.60% | 43% | 45.90% | 0.309352027 | 0.152101374 |
| CVD | No | 92.40% | 92.80% | 89.20% | 86.80% | 0.202690155 | 0.149714095 |
|  | Yes | 7.60% | 7.20% | 10.80% | 13.20% | 0.202690155 | 0.149714095 |

**STable 2** The marginal OR of IPTW + dual robustness was used as the supplementary result of the sensitivity analysis

| Variable | Level | 0 | <2 | ‘2-4’ | >4 | SMD_Unadj | SMD_IPTW |
| --- | --- | --- | --- | --- | --- | --- | --- |
| Age |  | 39 (19.6) | 48.1 (18.1) | 51.7 (16.7) | 54.6 (13.8) | 0.907740389 | 0.278432711 |
| BMI |  | 28.8 (7.8) | 28.9 (7.3) | 29 (6.8) | 28.9 (5.7) | 0.028696486 | 0.057484436 |
| Sex | Female | 52.70% | 62.80% | 48.40% | 40.80% | 0.445596282 | 0.020981382 |
|  | Male | 47.30% | 37.20% | 51.60% | 59.20% | 0.445596282 | 0.020981382 |
| Race | Mexican American | 11.60% | 11.50% | 8% | 3.90% | 0.720179459 | 0.168539447 |
|  | Non-Hispanic Black | 17.20% | 7.80% | 5.90% | 1.80% | 0.720179459 | 0.168539447 |
|  | Non-Hispanic White | 54.30% | 55.40% | 74% | 86.60% | 0.720179459 | 0.168539447 |
|  | Other Hispanic | 6% | 11.60% | 5.70% | 2.50% | 0.720179459 | 0.168539447 |
|  | Other Race - Including Multi-Racial | 11% | 13.80% | 6.30% | 5.20% | 0.720179459 | 0.168539447 |
| Smoke_status | No | 65.60% | 63.40% | 50.20% | 36.30% | 0.292493 | 0.061761 |
|  | Yes | 34.40% | 36.60% | 49.80% | 63.70% | 0.292493 | 0.061761 |
| Education | < High school | 27.40% | 17.30% | 13.20% | 11.20% | 0.434230423 | 0.139217751 |
|  | > High school | 49.40% | 61.10% | 65.70% | 65.90% | 0.434230423 | 0.139217751 |
|  | High school | 23.20% | 21.60% | 21.10% | 22.90% | 0.434230423 | 0.139217751 |
| Marital_status | Married/Living with Partner | 47.40% | 61.10% | 70.70% | 67.50% | 0.39513 | 0.124741 |
|  | Never married | 39.10% | 19.80% | 13.10% | 6.10% | 0.39513 | 0.124741 |
|  | Widowed/Divorced/Separated | 13.50% | 19% | 16.20% | 26.40% | 0.39513 | 0.124741 |
| Year | 2013-2014 | 32.80% | 27% | 29.70% | 30.40% | 0.183330132 | 0.050076476 |
|  | 2015-2016 | 35.20% | 32.20% | 33.50% | 34.10% | 0.183330132 | 0.050076476 |
|  | 2017-2018 | 32% | 40.80% | 36.70% | 35.50% | 0.183330132 | 0.050076476 |
| DM | No | 85.30% | 86% | 84.20% | 82.50% | 0.095794526 | 0.148923091 |
|  | Yes | 14.70% | 14% | 15.80% | 17.50% | 0.095794526 | 0.148923091 |
| Hypertension | No | 69.10% | 63.40% | 57% | 54.10% | 0.309352027 | 0.152101374 |
|  | Yes | 30.90% | 36.60% | 43% | 45.90% | 0.309352027 | 0.152101374 |
| CVD | No | 92.40% | 92.80% | 89.20% | 86.80% | 0.202690155 | 0.149714095 |
|  | Yes | 7.60% | 7.20% | 10.80% | 13.20% | 0.202690155 | 0.149714095 |

Fig. S1. Balance diagnosis plot (Figure S1, Love plot)
