# Supplementary figures and images for: Association between coffee consumption habits and non-alcoholic fatty liver disease in community-dwelling populations: data from the National Health and Nutrition Examination Survey 2013–2018
Source: Public Health Nutr. 2026 Feb 2;29(1):e35. doi: 10.1017/S1368980026101918 (PMC12951349; doi:10.1017/S1368980026101918)

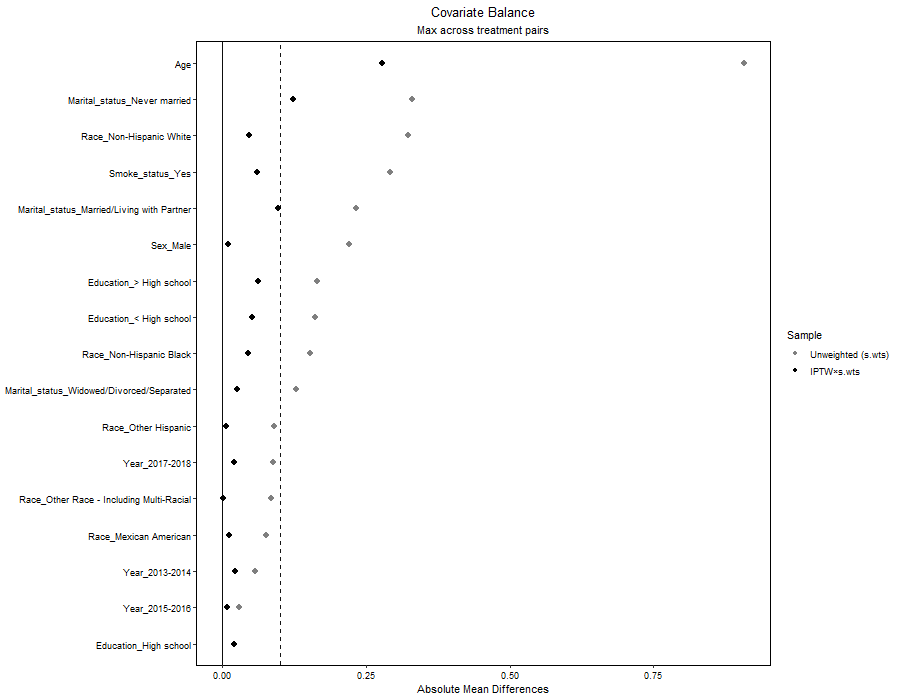

Supplement: Chen et al. supplementary material 2 — Chen et al. supplementary material [file S1368980026101918sup002.zip › SFigure 1.png]
